# Outcome of shock wave lithotripsy as monotherapy for large solitary renal stones (>2 cm in size) without stenting

**DOI:** 10.4103/0970-1591.70568

**Published:** 2010

**Authors:** Shanmugasundaram Rajaian, Santosh Kumar, Ganesh Gopalakrishnan, Ninan K. Chacko, Antony Devasia, Nitin S. Kekre

**Affiliations:** Department of Urology, Christian Medical College Hospital, Ida Scudder Road, Vellore, Tamil nadu, India

**Keywords:** Lithotripsy, renal calculi, SWL monotherapy, DJ stenting

## Abstract

**Purpose:**

To evaluate the outcome of shock wave lithotripsy (SWL) as monotherapy for solitary renal stones larger than 2 cm without ureteral stenting.

**Materials and Methods:**

Our retrospective study included patients with solitary renal radio opaque stones larger than 2 cm treated with SWL using electromagnetic Dornier Compact S lithotripter device (Wessling, Germany) for a period of 3 years (September 2002-2005). Stone clearance was assessed at 1 week, 1 month, and 3 months with plain X-rays of kidney, ureter, and bladder region, ultrasonography, and tomograms. Stone-free status, morbidity of the procedure, and fate of clinically insignificant residual fragments (CIRF) were studied. A stone-free state was defined as no radiologic evidence of stone. Success was defined as complete clearance + CIRF.

**Results:**

Fifty-five patients, aged 11–65 years (mean 49.8) underwent SWL. Of them, only two were children. Male-to-female ratio was 3:1. The stone size was 21–28 mm (average 24 mm). The mean number of shocks were 3732 (range 724–12,100) and average energy level was 14 kV (range 11–16 kV). The mean follow-up was 18 months (range 3–22 months). Over all, stone-free status was achieved in 50% and success in 81% at 3 months. Stone clearance was not affected by stone location. Stones <25 mm had better stone-free rate (54.16% vs. 28.5%) and lesser CIRF (14.6% vs. 28.5%) when compared to larger stones (>26 mm) (*P* = –0.10). Of 54 patients, 39 developed steinstrasse with mean length of 3.2 cm (range 1.4–6.2 cm) and only four required intervention. Effectiveness quotient (EQ) for SWL monotherapy for solitary renal stones more than 2 cm was 25.3%. The EQ for stones <25 mm and those more than 25 mm were 28.4% and 10% (*P* = –0.12), respectively. There was a lesser trend of difference between stones with size <25 and more than 25 mm. During the last review, the overall stone-free rate was 67.2%.

**Conclusions:**

SWL monotherapy was safe but significantly less effective for solitary renal stones larger than 2 cm. It can only be suggested to those who refuse surgical intervention. Pretreatment DJ stenting is not mandatory when closer follow-up is ensured.

## INTRODUCTION

The introduction of shock wave lithotripsy (SWL) for the treatment of renal stones by Chaussy *et al*. in 1980 has been the revolution of the century.[1] SWL has been proven to be an effective noninvasive treatment modality for most upper urinary tract calculi. The treatment of renal calculi is based on various factors such as size, location, composition of stones, and associated anatomical abnormalities.[2] Stone burden (size and number) is perhaps the single most important factor in determining the appropriate treatment modality for a patient with kidney calculi.[2]

Patients with calculi size between 10 and 20 mm are often treated with SWL as first-line management. Percutaneous nephrostolithotomy (PNL) is often the modality of treatment for stones of more than 20 mm in diameter. This is due to the higher retreatment rates and lower likelihood of achieving stone-free state with SWL in comparison of PNL.[3] Most would prefer to do pretreatment prophylactic DJ stenting when they prefer to treat larger renal stones (>2 cm) with SWL due to fear of having complications. In our department, as a policy we do not follow prophylactic DJ stenting even for larger renal stones since patients are closely followed during whole treatment session. This study is aimed at to assess the efficacy of SWL as monotherapy for larger renal stones and the safety of this therapy without prophylactic DJ stenting. We studied the outcome of SWL monotherapy in patients with solitary renal stones greater than 2 cm who opted for it after knowing the various options of treatment.

## MATERIALS AND METHODS

We analyzed the hospital records of patients who underwent SWL monotherapy with electromagnetic Dornier Compact S lithotripter (Wessling, Germany) for solitary radio-opaque nonstaghorn renal stones larger than 2 cm without stenting during a period of 3 years (September 2002–2005). Those who had congenital anomalies, renal failure, and those who underwent SWL following percutaneous nephrostomy, previous surgery, and stenting were excluded from the study. Pretreatment kidney, ureters, and bladder (KUB) plain films and intravenous urography were performed in all patients. Post-treatment follow-up ultrasonography, tomograms, and KUB plain films were used to monitor the fragmentation and clearance of fragments at 1-week, 1-month, and 3-month-period. Stone size was calculated by measuring the maximum dimensions of the stone in KUB plain films. All were treated in supine posture and underwent lithotripsy with a gradual incremental energy increase from 11 to 16 kV and at frequency varying from 70 to 120 min^−1^. We standardized our treatment protocol over last 2 years of the study period to 1500 shocks per session at frequency of 70 min^−1^at energy level of 14 kV as we saw better stone fragmentation and clearance with it.[4] The required number of shocks per session was decided on the basis of the observation of adequate stone fragmentation under fluoroscopy or ultrasonogram by the treating radiographer and physician. Adults were treated under sedoanalgesia using 50 mg pethidine and 25 mg promethazine. Children were treated under general anesthesia. All patients were advised to have fluid intake of about 2.5–3 L day^−1^. All were instructed to report even the minor complications after treatment and were kept under a close follow-up. In this study, complete clearance or stone-free state was defined as having no stone fragments at 3 months radiologically. Incomplete clearance was defined as having stone fragments of 5 mm or more in size. Clinically insignificant residual fragments (CIRFs) were defined as having stone fragments of 4 mm or less in diameter in asymptomatic nonstruvite patients. Successful outcome was defined as being stone free or having CIRF at 3 months. Unsuccessful outcome was incomplete clearance or failure of fragmentation at 4500 shocks. Efficiency quotient (EQ) was assessed by EQ = percentage stone free/(100% [1 treatment] + percentage requiring retreatment + percentage requiring auxiliary procedure) × 100%. All statistical analyses were performed using Statistical Package for the Social Sciences (SPSS 11.0) for windows. All categorical data were presented using frequencies and percentage. Associations between categorical variables were assessed using Chi-square test with Yates’ correction or Fisher’s exact test. A *P* value less than 0.05 was considered statistically significant. Phi/Cramer’s V correlation coefficient of trend of difference was also calculated.

## RESULTS

From January 2002 to September 2005, 792 patients having upper urinary tract stones underwent SWL in our institution. Fifty-five patients underwent SWL for larger solitary renal stones (>2 cm). The mean age of the patients was 49.8 years (range 11–65 years). Only two of them were children. Male-to-female ratio was 3:1. The stone size was 21–28 mm (average 24 mm). Forty-eight patients had stone size between 21 and 25 mm, and seven had size between 26 and 28 mm. All were followed up till they are stone free and during last review. The mean follow-up was 18 months (range 3–22 months). The mean number of shocks were 3732 (range 724–12,100), and average energy level was 14 kV (range 11–16 kV). All had complete stone fragmentation except one patient (1.8%) and he underwent PNL. Of the 55 patients, 32 (59.2%) had complete stone fragmentation with less than 6000 shocks and 14 (26%) with less than 9000 shocks in divided sessions.

Of the 55 patients who had treatment, 28 (50%) were stone free at 3 months, 17 (31%) and 10 (19%) had CIRF and incomplete clearance respectively. Overall successful outcome as defined in this study was observed in 81%.

Of 10 who had incomplete clearance, two were lost for follow-up, four underwent PNL, and four achieved stone-free status later. Of the 16 patients with CIRF, eight were lost for follow-up. Of the remaining eight patients, five expelled all the stone fragments during follow-up. Three had calculi remaining at the same size. No recurrence was noted in all those who achieved stone-free status at the last follow-up.

As denoted in the Table 1, the stone-free status, clinically residual fragments, and incomplete clearance were not different with respect to stone location. The difference was not statistically significant (*P* = -0.82). Patients with stones lesser than 25 mm size had better stone-free rate as compared to those who had stones larger than 25 mm (54.16% vs. 28.5%) However, the difference was statistically not significant (*P* = -0.10, Phi/Cramer’s V correlation coefficient of trend of difference was +0.22). CIRF was the same in both these groups. We observed that the stone-free status was almost the same irrespective of the stone location as denoted as follows: renal pelvis (51.6%), upper calyx (50%), middle calyx (53.3%), and lower calyx (53.3%). Effectiveness quotient for SWL monotherapy for solitary renal stones larger than 2 cm was 25.3%. EQ for renal pelvic, upper, middle, and lower calyceal calculi were 24.2%, 25%, 24.8%, and 27.5%, respectively [Table 2]. The EQ of stones with size smaller than 25 mm and those larger than 25 mm were 28.4% and 10%, respectively [Table 3]. There was a lesser trend of difference between the stone size and EQ (*P* = -0.12, phi/Cramer’s V difference was +024).

**Table 1:** Outcome of SWL in relation to stone location and size*

	No. of patients (%)					
	Total(%)	Stone-free status (%)	Incomplete clearance (%)	Residual fragment(s) (%)	Failure to fragmentation (%)	Success (%)
Location						
Pelvis	31 (60)	16 (51.6)	6 (19.3)	8 (25.8)	1 (3.3)	77.4
Upper calyx	6 (9)	3 (50)	1 (16.6)	2 (33.4)		83.4
Middle calyx	3 (4)	1 (33.3)		2 (66.7)		100
Lower calyx	15 (27)	8 (53.3)	3 (20)	4 (26.6)		79.9
Size (mm)						
21-25	48 (87)	26 (54.16)	7 (14.6)	14 (29.16)	1 (1.8)	83.32
26-28	7 (13)	2 (28.5)	3 (43)	2 (28.5)		57

* *P* > 0.05 for all.

**Table 2:** Outcome of SWL in respect to stone location*

Stone location	No. of patients	No. of stones retreated (%)	Mean No. of treatment sessions (range)	No. of stone free (%)	No. of auxiliary procedures (%)	No. CIRFs	Effectiveness quotient (%)
Renal pelvis	31	28 (90.3)	1.56 (1-5)	16 (51.6)	7 (22.5)	8 (25.8)	24.2
Upper calyx	6	4 (66.6)	2.78 (1-4)	3 (50)	2 (33.3)	2 (33.4)	25
Middle calyx	3	1 (33.7)	1.75 (1-3)	1 (33)	-	2 (66.7)	24.8
Lower calyx	15	12 (80)	3.4 (1-7)	8 (53.3)	2 (13.3)	4 (26.6)	27.5
Overall	55	45 (81.8)	3.1 (1-7)	28 (51.2)	11 (20)	10 (19)	25.3

* *P* > 0.05 for all

**Table 3:** Outcome of SWL in respect to stone size*

Stone size (mm)	No. of patients	No. of stones retreated (%)	Mean no. of treatment sessions	No. of stone free (%)	No. of auxillary procedures (%)	No. CIRFs (%)	Effectiveness quotient (%)
21-25	48	38 (79)	1.9 (1-5)	26 (54.16)	6 (12.5)	14 (29.16)	28.4
26-28	7	7 (100)	3.5 (2-7)	2 (28.5)	5 (71.4)	2 (28.5)	10

* *P* > 0.05 for all

After SWL, 39 of the 54 patients (72%) developed steinstrasse. Of them, 34 had type 1, 3 had type 2, and 2 had type 3 steinstrasse, respectively.[5] The mean length of steinstrasse was 3.2 cm (range 1.4–6.2 cm). The average duration for clearance of steinstrasse was 9.1 days (range 6–27 days). All were monitored radiologically using KUB X-rays and regular ultrasound examination to assess the clearance of steinstrasse and progression of hydronephrosis. Only 4 of 39 patients (10%) who developed steinstrasse needed intervention in the form of PCN and ureteroscopy for febrile Urinary Tract Infection (UTI) and failure of clearance of stone fragments. Two underwent PCN, one underwent URS, and one had both procedures. Two underwent SWL for lead fragments and had type 2 steinstrasse. One patient had acute urinary retention due to calculus obstruction and subsequently underwent cystolitholapaxy.

## DISCUSSION

SWL has become the standard initial treatment for most renal calculi since its introduction by Chaussy *et al*.[1] The role of SWL for larger renal stones is controversial. According to NIH consensus conferences recommendation, patients with stones larger than 2 cm, infected or not should be approached with PNL initially, followed if needed by Shock Wave Lithotripsy (SWL) due to high retreatment rates and the need for ancillary procedures.[6] However, many of the centers across the globe treat these patients with SWL monotherapy with good success rates.[7] A decade ago, the results of SWL monotherapy for solitary renal stones >2 cm were variable and stone-free rate was varying from 33% to 65%.[6] The advancement of technology and current expertise in SWL have yielded much higher stone-free rates.[7] In our study, 55 patients with solitary larger renal stones (>2 cm) underwent SWL monotherapy with electromagnetic Dornier compact S lithotripter (without DJ stenting). Success was achieved in 81% of the cases. Stone-free status at last review was seen in 67.2% of the patients in spite of the fact that a sizable number of patients (18%) were lost for further follow-up. It is important to note that among those who were lost for follow-up, many had CIRF. We had one case of failure to fragmentation (1.8%) and he underwent PNL later, and there were four cases of incomplete clearance who underwent PNL. The higher rate of retreatment and number lost for follow-up observed in our study would be due to varied distribution of patients from various parts of the country for whom stone-free status has to be achieved at limited period of stay?

### Success and stone-free status

Abe *et al*. reported 46% stone clearance and 54% residual fragments among 267 patients with renal stones with size between 20 and 30 mm in size in their series of 3024 patients treated with SWL monotherapy. All the patients had DJ stenting prior to SWL monotherapy. Their overall stone-free rate was 65.1%, and the success rate was 85.7% when they analyzed all the patients with stone size varying between 4 and > 30 mm.[8] Our study results are comparable to them as we achieved a stone-free rate of 67.2% and a success rate of 81% on stones more than 2 cm size, but without stenting. During the last decade, many studies had lower stone-free rate with SWL monotherapy for larger renal stones. Psihramis *et al*. reported on 94 renal stones larger than 2 cm treated with ESWL, with only 31 (33%) becoming stone free. Patients with multiple stones had a similar stone-free rate of 32%.[9] Lingeman *et al*. showed that the frequency of multiple treatments increased from 10% to 33% when SWL was used to treat stones of 1–2 cm and 2–3 cm, respectively. In addition, stone-free rate was only 34%, compared with 90% in PNL-treated patients.[10] With the advent of lithotripters with great efficacy, the results of shockwave monotherapy for larger renal stones has changed remarkably in the recent years. Shouman *et al*. has treated 24 children who had large solitary renal stones (25–35 mm) with SWL monotherapy without stenting.[7] Their stone-free rate was 83.3%. The stone-free rate observed in their series was remarkably high, probably due to the fact that pediatric ureters tend to expel larger stone fragments without difficulty. However, they have treated all their patients at higher energy level {average –17 kV (14–20 kV)}. We have achieved good stone fragmentation of larger renal stones at a lower energy level {average –14 kV (11–16 kV). Kurien *et al*. have shown that equivalent stone fragmentation and clearance as adults can be achieved in children with stone size less than 20 mm at lower shock rate and lower energy level.[4] Chacko *et al*. had shown in their study that slower rate of shock delivery has better fragmentation than faster rate in stones with size between 1 and 2 cm.[11] In our study, we have confirmed that with slower rate of shock delivery (70 shocks/min) has better stone fragmentation even for stone more than 2 cm.

### Is stenting necessary before SWL for larger renal stones?

The role of DJ stenting prior to SWL for large renal stones is controversial. DJ stenting prior to SWL monotherapy is often a prerequisite when treating renal stones of size more than 2 cm in order to get better clearance and avoiding complications. Low *et al*. compared 152 and 27 patients with small renal stones (<20 mm) who were treated without or with DJ stenting. There was no significant difference in stone-free rates at 1 month and 3 months (61% nonstented vs. 67% stented group) or in the retreatment rates (13.3% nonstented group vs. 14.8% stented group). Moreover, the incidence and severity of pain were similar groups. They concluded that placement of DJ stents for the purpose of improving stone-free rates, alleviating pain, or preventing ureteral obstruction in conjunction with SWL of solitary renal calculus <20 mm in diameter is unnecessary.[12] However, DJ stent has shown to increase the stone-free rates and reduce the complications due to ureteric obstruction and the need for percutaneous nephrostomy.

Libby *et al*. found that pretreatment placement of silicone ureteral stents reduced complications from 26% to 7% and auxiliary procedures from 15% to 6% when treating 283 kidneys with stone burden exceeding 25 mm with SWL.[13] Shouman *et al*.[7] has stressed that DJ stenting is not a prerequisite for success with SWL monotherapy even when treating larger stone bulk (25–35 mm). In their series, 25% of the 24 patients had complications related to steinstrasse and only one patient required intervention. Kumar *et al*. have shown that SWL monotherapy for renal stones without stenting even in solitary kidneys is safe.[14] In their series of 16 patients with solitary kidneys who underwent SWL monotherapy for renal stones between 5 and 15 mm without stenting, only one patient had complications due to obstruction of ureter.[14] In spite of conflicting reports on pretreatment stenting before SWL monotherapy, our study have emphasized the fact that pretreatment stenting is not mandatory for the success provided closer follow-up is ensured.

### Steinstrasse and complications

The incidence of steinstrasse is higher when SWL monotherapy is given for larger renal stones. Shouman *et al*.[7] have shown that steinstrasse occurred only in four patients (16.6%) in their study. Most of them have cleared the stone fragments without any problem and one underwent ureteroscopy.

In our study, even though 39 (72%) patients had steinstrasse following SWL, only 4 (10%) of them required intervention for complicated UTI and failure to clear. Most of the patients had type 1 steinstrasse and intervention was required only in those who had type 2 and 3 steinstrasse. The higher incidence of steinstrasse noted in the study probably is due to increased frequency of follow-up X-ray KUB taken at an earlier period and probably frequent treatment sessions within a short duration. Only two of those who had steinstrasse (type 2) were treated with SWL for leading fragments. Four patients had fever more than 38 °C in spite of negative urine cultures and all were managed conservatively. Many cleared their stone fragments within 4 weeks despite the fact that all of them received SWL monotherapy without stenting. As we did not do routine ultrasound examination immediately after SWL in every one, the incidence of renal subcapsular hematoma is unknown in our series. Early recognition of complications and close monitoring of those patients who are at risk of increased steinstrasse formation like stone size more than 2.5 cm is mandatory to avoid untoward results, especially when no urinary diversion maneuvers like DJ stenting is being practiced.

As observed in this study, stone clearance was not different irrespective of stone location, especially lower calyceal stones. This could be due to selection bias and strict adherence to the instructions regarding fluid intake, postural therapy, etc. We believe that these recommendations could play a major role in achieving better stone-free status.[15] The strengths of this study are: rapid intensive treatment schedule and closer follow-up in a situation in which patients are staying close to the hospital for the whole treatment schedule. The limitations in our study were: (1) small number of patients been treated with SWL monotherapy, (2) heterogenous group of patients including children, and (3) heterogenous treatment protocol followed apart from the usual limitations of a retrospective study. When EQ was calculated, SWL monotherapy was not effective for larger renal stones. The low-efficiency quotient seen in this study could be due to higher retreatment rate observed. This increased retreatment was followed to achieve maximal stone clearance within a limited period by pulverizing the stone fragments further to facilitate the stone expulsion, after adequate stone fragmentation. However, with this small population, we were able to achieve the same stone-free and success rates comparable to larger series.[7,8,12] A comparison study between PNL and SWL for larger renal stones comparing stone-free rate, retreatment rate, economics of treatment, and complications after standardization of treatment schedule is needed in Indian subcontinent to say the final verdict emphatically.

## CONCLUSIONS

In the present era of rapidly advancing endourology and clear-cut guidelines on the role of PNL, the plan of SWL seems to be in prophecy. From the patient perspective, the advantage of SWL is its noninvasive nature. This paper is presented to assess the role of SWL in the treatment of larger stones.

After evaluating the patients treated with SWL monotherapy without stenting for solitary renal stones of more than 2 cm size, we found that SWL was safe but less effective. Stone-free rate was better with the stones <25 mm when compared to larger stones more than 25 mm size. SWL monotherapy should only be suggested for those who refuse surgical intervention for larger renal stones (>2 cm in size). This paper also confirmed that routine DJ stenting is not mandatory for the success if stringent follow-up and proximity to medical facilities ensured.

## References

[1] Chaussy C, Brendel W, Schmiedt E (1980). Extracorporeally induced destruction of kidney stones by shock waves. Lancet.

[2] Motola JA, Smith AD (1990). Therapeutic options for the management of upper tract calculi. Urol Clin North Am.

[3] Lingeman JE, Coury TA, Newman DM, Kahnoski RJ, Mertz JH, Mosbaugh PG (1987). Comparison of results and morbidity of percutaneous nephrostolithotomy and extracorporeal shock wave lithotripsy. J Urol.

[4] Kurien A, Symons S, Manohar T, Desai M (2009). Extracorporeal shock wave lithotripsy in children: Equivalent clearance rates to adults is achieved with fewer and lower energy shock waves. BJU Int.

[5] Coptcoat MJ, Webb DR, Kellet MJ, Whitfield HN, Wickham JEA (1988). The steinstrasse: A legacy of ESWL. Eur Urol.

[6] Renner CH, Rassweiler J (1999). Treatment of renal stones by extracorporeal shock wave lithotripsy. Nephron.

[7] Shouman AM, Ziada AM, Ghoneim IA, Morsi HA (2009). Extracorporeal shock wave lithotripsy monotherapy for renal stones >25 mm in children. Urology.

[8] Abe T, Akakura K, Kawaguchi M, Ueda T, Ichikawa T, Ito H (2005). Outcomes of shockwave lithotripsy for upper urinary-tract stones: A large-scale study at a single institution. J Endourol.

[9] Psihramis KE, Jewett MA, Bombardier C, Caron D, Ryan M (1992). Lithostar extracorporeal shock wave lithotripsy: The first 1,000 patients. Toronto Lithotripsy Associates. J Urol.

[10] Lingeman JE, Lingeman JE, Smith LH, Woods JR, Newman DM (1989). Non-staghorn renal calculi. Urinary Calculi.

[11] Chacko J, Moore M, Sankey N, Chandhoke PS (2006). Does a slower treatment rate impact the efficacy of extracorporeal shock wave lithotripsy for solitary kidney or ureteral stones?. J Urol.

[12] Low RK, Stoller ML, Irby P, Keeler L, Elhilali M (1996). Outcome assessment of double-J stents during extracorporeal shockwave lithotripsy of small solitary renal calculi. J Endourol.

[13] Libby JM, Meacham RB, Griffith DP (1988). The role of silicone ureteral stents in extracorporeal shock wave lithotripsy of large renal calculi. J Urol.

[14] Kumar S, Sakthivel A, Chacko KN, Kekre NS, Ganesh G (2006). Shock wave lithotripsy in solitary functioning kidneys: Is prophylactic stenting necessary?. Urol Int.

[15] Tolon M, Miroglu C, Erol H, Tolon J, Acar D, Bazmanoglu E (1991). A report on extracorporeal shock wave lithotripsy results on 1,569 renal units in an outpatient clinic. J Urol.

